# Morphological and molecular analysis of cryptic native and invasive freshwater snails in Chile

**DOI:** 10.1038/s41598-019-41279-x

**Published:** 2019-05-24

**Authors:** Gonzalo A. Collado, Marcela A. Vidal, Karina P. Aguayo, Marco A. Méndez, Moisés A. Valladares, Francisco J. Cabrera, Luis Pastenes, Diego E. Gutiérrez Gregoric, Nicolas Puillandre

**Affiliations:** 1 Departamento de Ciencias Básicas, Facultad de Ciencias, Universidad del Bío-Bío, Chillán, Chile; 2 Grupo de Biodiversidad y Cambio Global, Universidad del Bío-Bío, Chillán, Chile; 3 Laboratorio de Genética y Evolución, Facultad de Ciencias, Universidad de Chile, Santiago, Chile; 4 Universidad Tecnológica de Chile INACAP, Chillán, Chile; 5 Departamento de Biología y Química, Facultad de Ciencias Básicas, Universidad Católica del Maule, Talca, Chile; 6 División Zoología Invertebrados, Museo de La Plata, Facultad de Ciencias Naturales y Museo, Universidad Nacional de La Plata, La Plata, Buenos Aires, Argentina; 7 Consejo Nacional de Investigaciones Científicas y Técnicas (CONICET), CCT La Plata, Buenos Aires, Argentina; 8 Institut Systématique Evolution Biodiversité (ISYEB), Muséum national d’Histoire naturelle, CNRS, Sorbonne Université, EPHE, 57 rue Cuvier, CP 26, 75005 Paris, France

**Keywords:** Taxonomy, Biodiversity

## Abstract

Species delimitation in minute freshwater snails is often difficult to perform using solely shell morphology. The problem intensifies when invasive species spread within the distribution range of morphologically similar native species. In Chile, the Truncatelloidean snails are represented by the native genera *Heleobia* and *Potamolithu*s plus the invasive mudsnail *Potamopyrgus antipodarum*, which can easily be confused. Using an integrative approach, we performed molecular phylogenetic analysis and studied reproductive and morphological features to identify superficially similar forms inhabiting the central area of the country. Truncatelloidean snails were identified in 40 of 51 localities sampled, 10 containing *Potamopyrgus antipodarum*, 23 *Heleobia* and 7 *Potamolithus*. Based on these results and previously published data, the known distribution of the mudsnail in Chile encompasses 6 hydrological basins, including 18 freshwater ecosystems. The finding of the mudsnails in several type localities of native species/subspecies of “*Heleobia*” that were not find *in situ* suggests species replacement or significant extinction of native fauna, a hypothesis supported by the restudy of type material that shows that endemic forms belong to the genus *Potamolithu*s. This study shows the usefulness of integrative taxonomy not only resolving complex taxa with cryptic morphology but also measuring the extent of an ongoing invasion.

## Introduction

Non-native species can generate a range of effects on ecosystems invaded. One of the most serious impacts on the environment is the threat to endemic biodiversity, which may be affected through population demise, rarefaction, species displacement and even extinction^1–7^. In some cases, biological invasions may be difficult to recognize due to the introduction of cryptic species^8^, a problem that intensifies when invasive species colonize ecosystems located within the known distribution range of morphologically similar native species, which may lead, in turn, to unfortunate misidentifications^9^.

The gastropods of the superfamily Truncatelloidea include minute freshwater snails that are difficult to identify due to small size, substantial intraspecific variability and convergence of external shell features^9–12^. In Chile, these snails are represented by two native genera: *Heleobia* Stimpson, 1865 (Cochliopidae), with about 30 species, and *Potamolithus* Pilsbry, 1896 (Tateidae species), composed by *Potamolithus* *australis* Biese, 1944 and *Potamolithus* *santiagensis* (Biese, 1944)^13–22^. A third representative is the mudsnail *Potamopyrgus antipodarum* (Gray, 1843) (Tateidae), native to New Zealand^23,24^, a highly invasive species the morphology of which closely resembles snails of the two Chilean endemic genera^25,26^. Besides these taxa, the mudsnail can also be confused with other Tateidae as well as other species belonging to different gastropod families, including Cochliopidae, Amnicolidae, Bithyniidae, Hydrobiidae, and Lithoglyphidae^27–32^.

The morphological similarity of the mudsnail with native snails has led to misidentifications in invaded habitats. For instance, in Garden Lakes, USA, the native *Tryonia porrecta* (Mighels, 1845) was incorrectly identified as the mudsnail^31^ whereas in Nordic areas from Europe, some species of *Bithynia* Leach, 1818 are also superficially similar in morphology to this species^29^. The mudsnail also resembles the snail *Pyrgophorus platyrachis* Thompson, 1968, native to Florida and subsequently introduced in Singapore and the Middle East^9,11,12^. Identification errors can even lead to the description of a cryptic invasive species as a new species, making the correct identification of the invasive species, and consequently, the invasion, overlooked^25,33,34^. Perhaps one of the more striking examples of this occurred at the end of the 19th century when the mudsnail was described from the Thames estuary (UK) as *Potamopyrgus jenkinsi* (Smith, 1889), an overlooked biological invasion that was finally confirmed almost a century later^23,33,35^. Similarly, in Australia and Tasmania the mudsnail was also erroneously recognized as *Potamopyrgus niger* (Quoy and Gaimard, 1835) for a long time^33^. In Chile, the presence of the mudsnail was first reported in 2014 and later in 2016 but the species was introduced earlier since it was confused with native species of the genus *Heleobia* in 2011^18,25,26^. Accurate identification of freshwater snails in the Southern Cone of South America becomes even more difficult considering that native species of *Potamolithus* have been confused with *Heleobia*^18,22,36–38^, for example “*Heleobia*” *hatcheri* (Pilsbry, 1911) and “*Heleobia*” *santiagensis*.

The invasive populations of the mudsnail can reach very high densities in the invaded ecosystems^39–43^. Investigations of the effect of this species on these environments have shown both positive and negative relationships with the invertebrate taxa^44^. For example, some studies have demonstrated the absence of significant changes in local communities with respect to biomass and biodiversity, as well as decrease in population abundances and local extinctions of some species^22,41,45–49^. However, other studies have found an increase in densities of macroinvertebrates, as well as an increase in family richness and diversity^44,50,51^.

Molecular analyses based on DNA sequences are a powerful tool for accurate identification of invasive species^52,53^, including freshwater snails^25,31,34,54–56^. These studies can be coupled to traditional morphological investigations in an integrative way. The objective of this study was to identify cryptic native and invasive snails of the superfamily Truncatelloidea distributed in central Chile, including samples collected from six type localities of native species, using molecular and morphological analysis. We also reexamined the type specimens of the taxa originally described under the genus *Littoridina* Souleyet, 1852^10,13–18^ in the study area to investigate if this represents original misidentifications or possible extinctions of native fauna.

## Results

### Taxa identification

**Figure 1:**
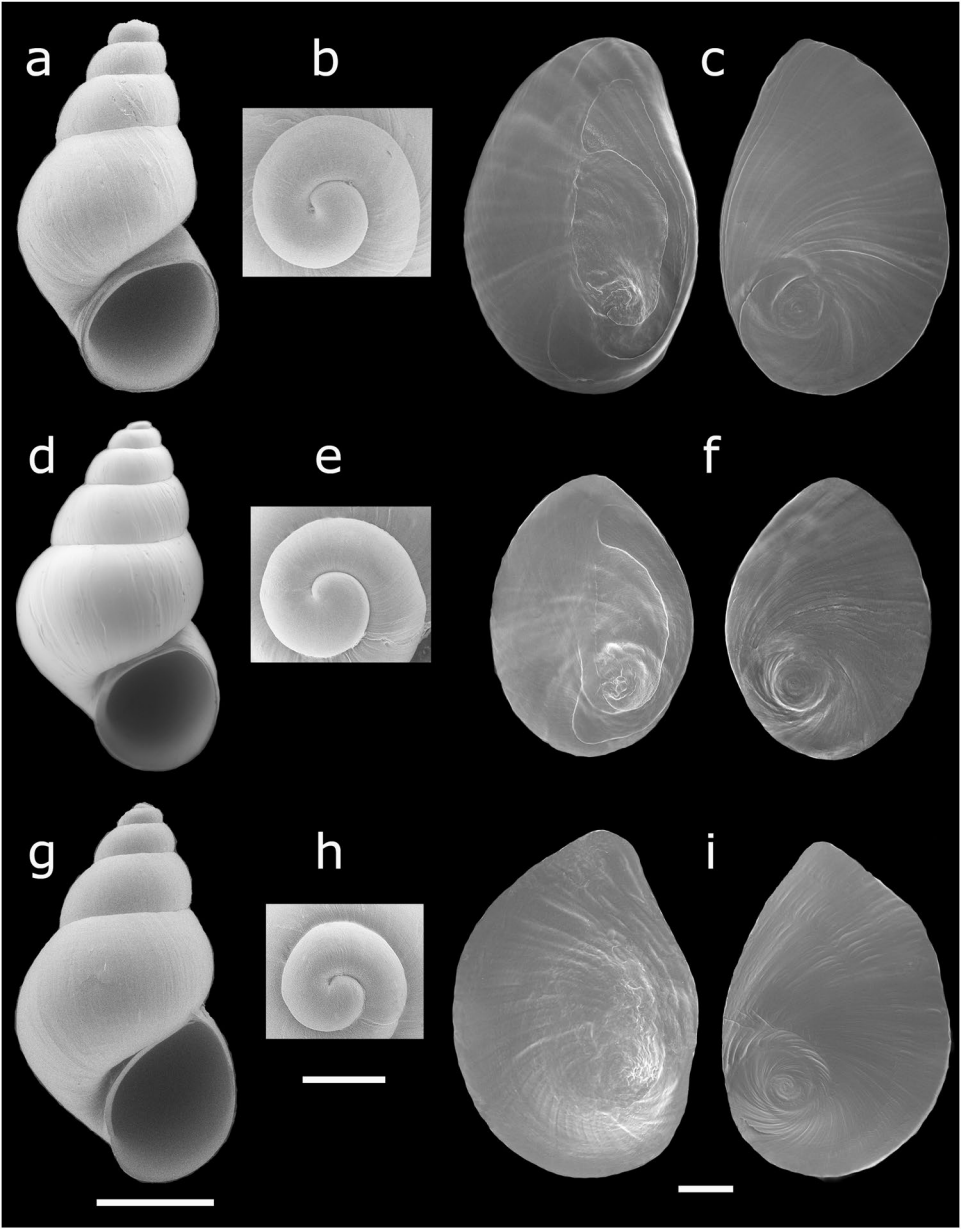
Images of cryptic truncatelloidean freshwater snails found in central Chile. Shell (**a**,**d**,**g**), protoconch (**b**,**e**,**h**) and operculum (**c**,**f**,**i**) of *Potamopyrgus antipodarum* (**a**–**c**), *Potamolithus* (**d**–**f**) and *Heleobia* (**g**–**i**). Scale bar: **a**,**d**,**g** = 1 mm; **b**,**c**,**e**,**f**,**h**,**i** = 200 µm. (Images: Gonzalo A. Collado).

**Figure 2:**
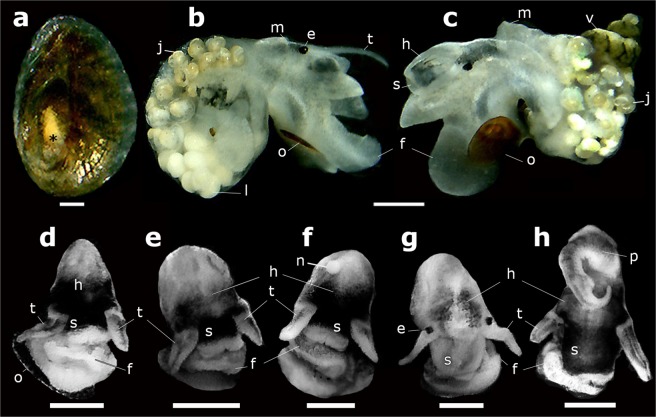
Operculum and soft body of invasive and native Chilean truncatelloidean snails. (**a**–**d**) *Potamopyrgus antipodarum*. (**a**) Operculum (external view) of an adult specimen showing a conspicuous white smear (asterisk). (**b**,**c**) Soft body of an adult specimen (right and left side, respectively) after dissection to show shelled juvenile within brood pouches. (**d**) Anatomy of the anterior region of the body. (**e**–**h**) Native snails. (**e**,**f**) Females of *Potamolithus* sp. from Lo Carreño. (**g**) Female of *Heleobia* sp. from El Yali. (**h**) Male of *Heleobia* sp. from Los Molles. Abbreviations: e, eye; f, foot; h, head; j, juvenile; l, larvae; m, mantle; n, nuchal node; o, operculum; p, penis; s, snout, t, tentacles; v, visceral mass. Scale bar: (**a**) = 200 µm; (**b**,**c**) = 1 mm; (**d**–**h**) = 500 µm. (Plate of photographs: Gonzalo A. Collado)

**Figure 3:**
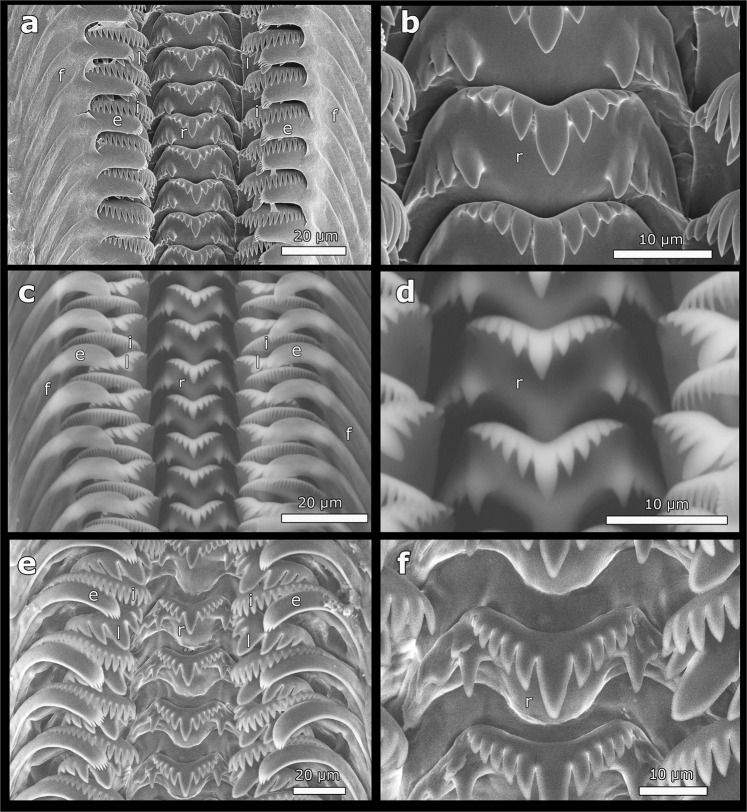
Radular morphology of cryptic truncatelloidean freshwater snails found in central Chile. Portion of the radular ribbon (left) and central radular teeth (right). (**a**,**b**) *Potamopyrgus antipodarum*. (**c**,**d**) *Potamolithus* sp. (**e**,**f**) *Heleobia* sp. Note the external marginal tooth (**e**), flange (**f**), internal marginal tooth (i), lateral tooth (l) and rachidian tooth (r). (Images: Gonzalo A. Collado).

Of 51 localities sampled, the preliminary morphological examination identified truncatelloidean snails in 40 of them (Table S1). The shell, superficially similar among populations (Fig. 1a,d,g), precluded an objective and prompt identification of taxa in the field. A close examination of the protoconch (Fig. 1b,e,h) and operculum (Fig. 1c,f,i) allowed us to differentiate the mudsnail from *Heleobia* and *Potamolithus* snails and also to distinguish between these two endemic genera, but with greater difficulty. The protoconch showed differences in length among genera, being longer in the mudsnail. The operculum, corneous, paucispiral and with eccentric nucleus in all populations studied, showed differences among genera regarding the color and the presence/absence of a white smear (Fig. 2a). Reproductive features showed some particular differences in secondary characters, sexuality and reproductive strategies that also allowed differentiating among genera (Fig. 2b–h). In all populations examined the radula is taenioglossan (Fig. 3a–f), with seven teeth on each row, including two marginal teeth (external and internal) plus a lateral tooth placed on each side of the central (rachidian) tooth (Fig. 3a,c,e); the general formula is 3–1–3. This structure allowed identifying snails to the family level according to differences provided by the central and external marginal teeth. A summary with the main morphological differences of the samples studied are shown in Table 1.

**Table 1:** Morphological features and reproductive strategies of truncatelloidean snails sampled in Chile.

Genus/species	Shell shape	Protoconch	Operculum	Radula		Brood pouches	Sexuality**	Nuchal** node
				CTF	EMT			
*Potamopyrgus antipodarum*	Ovate-conic; thin	Large; Whorls: 1^1/4^	Solid; bright brown/orange; with white smear	[(3 − 5) − 1 − (3 − 5)]/3 − 3	Whit flange well developed	yes	♀	No
*Potamolithus*	Ovate-conic; relatively thick	Median; Whorls: 1^1/8^	Thin; light brown, translucent	[(3 − 5) − 1 − (3 − 5)]/3 − 3	Whit flange well developed	No	♀*	Some populations
*Heleobia*	Ovate-conic; thin	Small; Whorls: nearly 1	Thin; gray, translucent, with a light brown central area	[(4 − 7) − 1 − (4 − 7)]/1 − 1	Long and thin; flange barely developed	No	♂, ♀	No

CTF: Central tooth formula; EMT: external marginal teeth; *: males were recorded in a single population; **: for a specific locality refer to Table S1.

Specimens of *Potamopyrgus antipodarum* were found in 10 sites, *Heleobia* spp. in 23 and *Potamolithus* spp. in 7. In no locality were all three genera found co-distributed. In all the localities where *Heleobia* occurs, no other truncatelloidean snail was found. *Potamopyrgus antipodarum* and *Potamolithus* live in syntopy in two localities. *Heleobia* populations were not identified at the species level due to the variability of morphological characters, the finding of putative new species and the fact that only mudsnails were found in six type localities of endemic species ascribed to this genus (Table S1). With the exception of *Potamolithus santiagensis* from El Yeso Spring and Lo Carreño^22^ native populations of this genus could not be identified to the species level because the samples are morphologically variable, potentially representing new species. In addition, our samples of *Potamolithus* populations were morphologically differentiable of the holotype of *Potamolithus australis*, a species that besides inhabit more than 800 km south of the southernmost site of the present study^13^.

**Taxonomic Account**

Class Gastropoda Cuvier, 1797

Superfamily Truncatelloidea Gray, 1840

Family Tateidae Thiele, 1925

Genus *Potamopyrgus* Stimpson, 1865

*Potamopyrgus antipodarum* (Gray, 1843)

#### Diagnosis

Shell shape ovate to elongated conical (Fig. 1a), with up to six to seven whorls, smooth, suture deep. Shell colors vary from grayish and dark brown to light brown. Aperture oval, sometimes with external lip thickened. Protoconch large, 1^1/4^ whorls (including nucleus) (Fig. 1b). Body colors vary from grayish-white or gray to grayish-black or black. Operculum bright orange-brown, ellipsoid, relatively thick, with a central muscle insertion area to the right of the nucleus and another long and narrow attachment region extending close to the inner margin (Fig. 1c); the central area encloses a conspicuous white smear (Fig. 2a). Females (Fig. 3b–d) ovoviviparous, containing embryos or shelled juveniles in the brood pouches (Fig. 2b,c). Males were not observed. Central tooth of the radula with three to five lateral cusps on each side of a larger median cusp and three pairs of basal cusps, which decrease in size distally (Fig. 3a,b). Flange well developed on the external marginal teeth, leaving free only the distal section of them.

#### Distribution

Eighteen localities in Central Chile, considering the 10 sites reported in the present study (Table S1) plus eight documented previously^22,25,26^. Región de Coquimbo: Huentelauquén (in the northern sector of the town), La Brunina, Consuelo Stream, Illapel River, Choapa River, Camisas Stream, Limahuida Stream; Salamanca (Chalinga River), Cunlagua (Chalinga River), Zapallar (Chalinga River), Canal Zapallar; Región de Valparaíso: Canal La Laja, Romeral Stream, Chincolco; Región Metropolitana: Dehesa Stream, Parque O’Higgins Spring, El Yeso Spring; Región de O’Higgins: Canal Lo Carreño. In El Yeso Spring and Lo Carreño mudsnails coexist with *Potamolithus santiagensis*^22^.

Genus *Potamolithu*s Pilsbry, 1896.

#### Diagnosis

Shell shape ovate to elongated conical (Fig. 1d), relatively thick, with up to five to six whorls, with very thin axial ribs, suture deep. Shell colors vary from light to dark brown. Aperture oval-rounded, outer lip slightly thick. Protoconch is 1^1/8^ whorls (Fig. 1e). Body colors vary from white to grayish-white or grayish-black. Operculum oval (Fig. 1f), light brown, translucent, thin, without white smear. Females (Fig. 2e,f) of several populations with nuchal node (Fig. 2f), without brood pouches (oviparous). Males having simple penis (without ornamentations), grayish-white. Radula of *Potamolithu*s (Fig. 3c,d), including external marginal teeth, as in mudsnail.

#### Distribution

*Potamolithus* snails were collected in seven localities in the present study (Table S1). Although we could not find topotype specimens of *Heleobia choapaensis* (Biese, 1944), *Heleobia choapaensis albolabris* (Biese, 1944), *Heleobia bruninensis* (Biese, 1944), *Heleobia compacta* (Biese, 1944) and *Heleobia* *choapaensis minor* (Biese, 1944) in ecosystems from the Choapa River basin, where these forms were originally described under the genus *Littoridina*^13,14^, the protoconcha of respective type specimens housed at the MNNHCL suggests that they belong to *Potamolithus*. The same was revealed for *Heleobia santiagensis* (Biese, 1944) from Dehesa Stream in the Maipo River basin. In Chile, the distribution records of the genus encompass from the Región de Coquimbo to the Región de Los Lagos according to our results and previously published data^13,14,22^ (Table S1). *Potamolithus santiagensis* occurs in Dehesa Stream, El Yeso Spring, Lo Carreño and El Colorado according to the present study, Biese^13,14^ and Collado *et al*.^22^. *Potamolithus* *australis* inhabits Puerto Chico in Llanquihue Lake, its type locality^13^, but this need to be confirmed^57^.

Family Cochliopidae Tryon, 1866

Genus *Heleobia* Stimpson, 1865.

#### Diagnosis

Shell shape ovate to elongated conical (Fig. 1g), thin, with up to six to seven whorls, with very thin axial ribs, suture deep. Shell colors vary from light brown-translucent to grayish. Aperture oval to ellipsoid, outer lip thin. Protoconch ca. 1 whorl (Fig. 1h). Body colors vary from light brownish-yellow to gray, grayish-white or grayish-black. Operculum oval to ellipsoid (Fig. 1i), thin, translucent, with a faint light brown central area, without white smear. Females (Fig. 2g) without brood pouches (oviparous), nuchal node absent. Males having penis with apocrine glands or lobes (Fig. 2h). Central tooth of the radula with four to seven lateral cusps on each side of a larger median cusp and one pair of basal cusps (Fig. 3e,f). External marginal teeth large, thin and free almost from the base (with flange poorly developed).

#### Distribution

**Figure 4:**
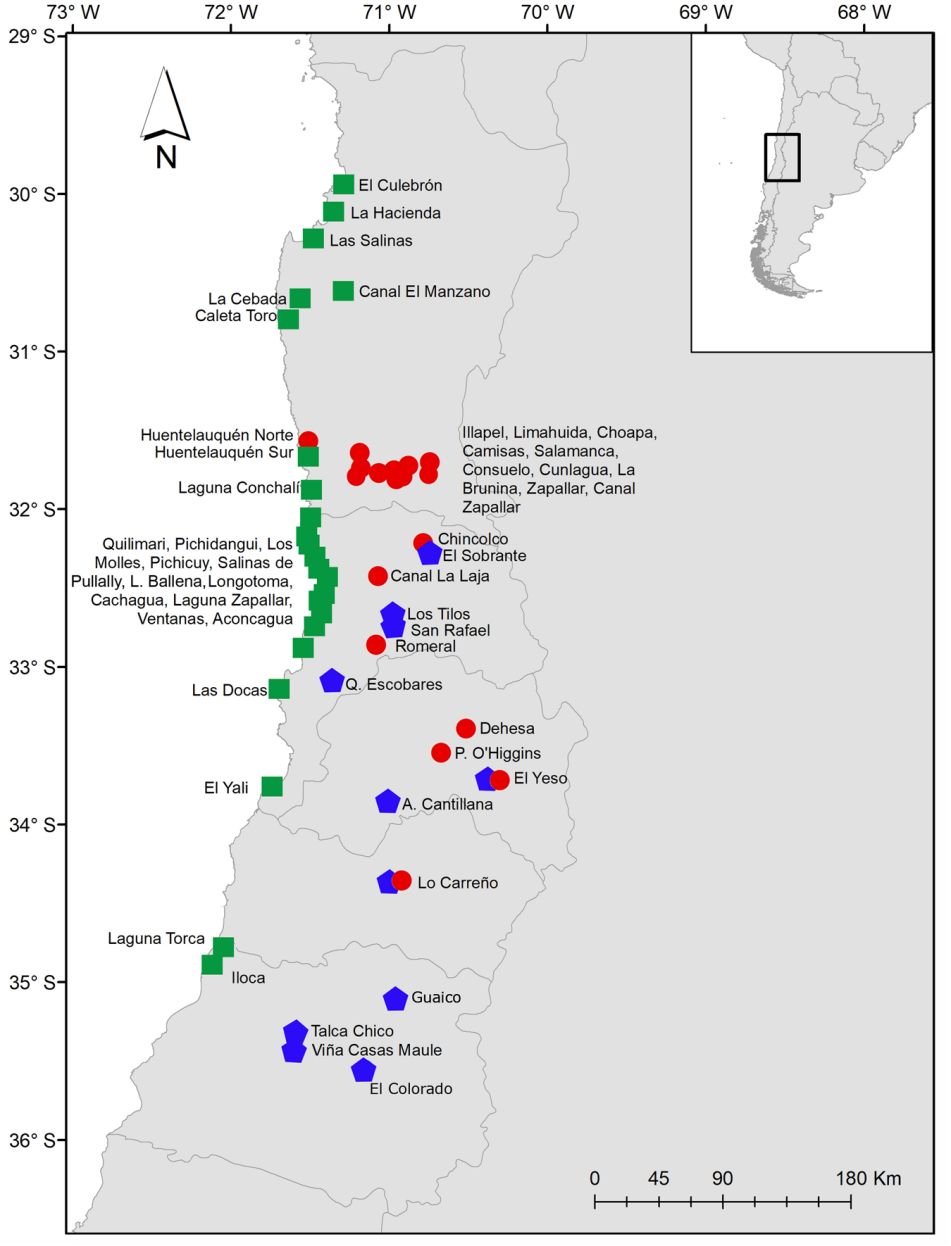
Known distribution records of the truncatelloidean taxa in central Chile according to the present study and other sources^22,25,26^. The red circles correspond to records of the invasive mudsnail *Potamopyrgus antipodarum*. Records of the native populations of the genera *Heleobia* and *Potamolithus* are indicated by green squares and blue pentagon, respectively. The map was created using ArcGIS software (www.arcgis.com/index.html). (Map: G.A. Collado).

In Chile the known distribution of the genus *Heleobia* encompass from the Región de Arica y Parinacota to the Strait of Magellan^13–21,58,59^. In the present study, snails were collected in 23 localities (Table S1). No case of syntopy was detected with the other taxa collected in the localities studied. The known distribution records of the truncatelloidean taxa in central Chile^22,﻿25﻿,26^, are shown in Fig. 4.

### Molecular analysis

**Figure 5:**
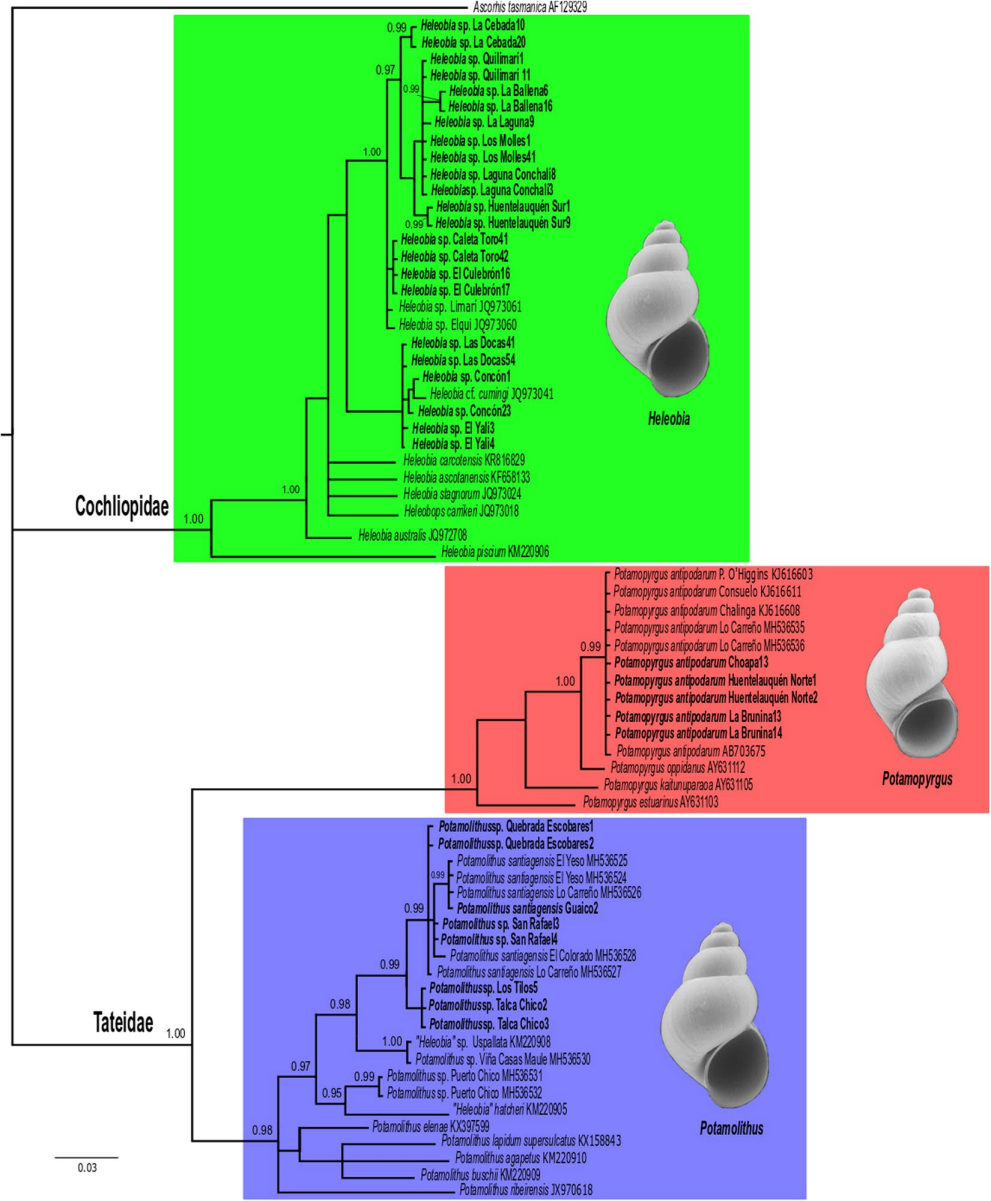
The Bayesian tree of Chilean truncatelloidean snails. Original sequences of specimens used in the present study are highlighted in bold. Posterior probabilities (>0.94) values are indicated at the nodes. Numbers after taxa names indicate GenBank access numbers^20–22,25,32,38,87,101–106^ (Table S2).

The Bayesian analysis using the *COI* gene recovered our original sequences into two main strongly supported clades, Cochliopidae (BPP = 1.00), including populations assigned to the genus *Heleobia*, and Tateidae (BPP = 1.00), composed by species/populations assigned to the genera *Potamolithus* (BPP = 0.98) and *Potamopyrgus* (BPP = 1.00), the latter represented by *Potamopyrgus antipodarum* (BPP = 0.99) (Fig. 5). Using our original *COI* sequences, divergence levels (*p-*distance) among groups were 15.1% between *P*. *antipodarum* and *Potamolithus*, 19.9% between the former and *Heleobia* and 17.8% between the latter and *Potamolithus*. The average *p-*distance within groups were 0.0% in *P*. *antipodarum*, 0.6% in *Potamolithus* and 2.5% in *Heleobia*.

## Discussion

In the present study, molecular, morphological and microstructural data were used to evaluate the occurrence of cryptic populations of native and non-native freshwater snails of the superfamily Truncatelloidea in central Chile. The data obtained show that the mudsnail *Potamopyrgus antipodarum* is widespread in the sampling area. This is a significant finding regarding conservation since the known distribution range of this species encompasses type localities of five native species or subspecies of *Heleobia* described from the Choapa River basin^13,14^, which are: *Heleobia choapaensis* (Choapa River), *Heleobia choapaensis albolabris* (Consuelo Stream), *Heleobia bruninensis* (La Brunina), *Heleobia compacta* and *Heleobia* *choapaensis minor* (Zapallar). Regarding these localities, it is important to emphasize two aspects, one is that considering the types specimens reexamined, Biese (1944) probably misidentified *Potamolithus* specimens and named the species under the genus *Littoridina* (currently assigned to *Heleobia*), and second, we showed that the mudsnail invaded these localities and apparently now there would be no native species inhabiting them. Similarly, in Dehesa Stream, type locality of *Heleobia santiagensis*^13,14,18,22^, only mudsnails have been found in different years of sample collections (2011, 2014, 2015, 2017)^18,22,25^, including the present study. The finding that the Biese’s “*Heleobia*”^13,14^ above-mentioned are *Potamolithus* raises the possibility that the mudsnail has replaced or extinguished native snails in several type localities. The occurrence of *Physella acuta* (Draparnaud, 1805) in the Choapa River basin^34^ and Dehesa Stream (unpublished data), another non-native freshwater snail in Chile^34^, could have contributed to the decline (or extinction) of the native snail populations. In El Yeso Spring and Lo Carreño, where *Physella acuta* has not been found, mudsnails are syntopic with *Potamolithus*, and although not evaluated, much more abundant so it is likely competition in some niche dimensions between them. Species displacement, or even extinction of native fauna in central Chile is plausible considering that after the introduction of the mudsnail to the Snake River drainage (U.S.), five species of native mollusks were officially listed as “threatened” or “endangered”, in part due to the proliferation of the mudsnail^41^. Besides, this species has also been associated with decline in settlement of native invertebrates^41,46,49^.

Due to its high invasive power, the mudsnail has been considered as one of the most widespread invasive aquatic invertebrates in the world^60,61^. In line with this status, ongoing research on this species have shown a rapid expansion in central Chile from 2011^18,25^, which can lead to serious consequences for the natural heritage of the country. It is clear that more sampling and molecular work is required in the future, especially outside of our study area, to better determine the prevalence, distribution and taxonomy of native taxa. Moreover, the present study also allowed extending the distribution of the genus *Potamolithus* hundreds of kilometers northward. Similarly, although mudsnail occurs in typical environments from which “*Heleobia*” species have been previously recorded in Chile^13,14,18^, several localities of occurrence were added here. This is also significant for conservation purposes since several species of this genus have been classified as vulnerable or critically endangered in Chile^62–65^. On the other hand, it is important to note that the mudsnail is an intermediate host of digenetic trematodes^66–68^, being able to transport parasites to non-native ecosystem^68^ whereby they could potentially affect native final vertebrate hosts, a potential problem not studied in Chile. Non-native disease vector, in addition to direct competition and displacement of endemic fauna could be one mechanism by which the invasive species places these taxa at risk. However, the mudsnail tend to finely partition the habitat regarding vegetation zones and depth^66–69^ so competition with endemic invertebrate could be, in part, diminished.

Different traits may explain the invasive success of the mudsnail^70^. The species is a generalist, can tolerate a wide range of environmental conditions^39,71,72^ and may achieve an astonishingly high reproductive rate. The latter is favored by the fact that invasive populations are composed almost exclusively by parthenogenetic females that can brood and release a lot of snails several times a year^23,41,73,74^. It has been estimated that under ideal conditions, a single female can produce 3.125 × 10^8^ offspring in six broods per year beginning with 50 offspring produced per female^41^. Besides, since a single female may establish a new invasive population^71,75^, it is not surprising that the species has spread quickly in Chile because until now only females have been found in the country^25,26^, present study. This may be also favored by a high dispersal capacity of the species, being able to use natural and non-natural vectors^27,40,71,76–78^. However, the mechanism of introduction and spreading of the species in Chile remains unknown.

The early detection of invasive species is fundamental to develop measures to mitigate its invasion and subsequent establishment in new ecosystems^79^. The method used here to evaluate the presence of the mudsnail and morphologically similar cryptic truncatelloidean taxa may be applied in other geographical areas since members of this superfamily are widespread around the world^10,80^. This would constitute the first step towards taking appropriate control measures to treat a new invasion or at best achieve timely eradication.

## Methods

### Specimen collection

We prospected 51 freshwater ecosystems in Central Chile between 2015 and 2017 (Table S1), including brackish waters, swamps, reservoirs, rivers, streams, springs, ponds and irrigation canals. Snail specimens were sampled using a manual sieve (1 mm mesh) and preserved in absolute ethanol. A few specimens with uncertain taxonomic status sampled previously^18,22^, in press were further studied to assign the species to genera. The first author was authorized to collect freshwater snails in Chile (Subsecretaría de Pesca y Acuicultura, Ministerio de Economía, Fomento y Turismo, República de Chile, Resolution No. 3285).

### Morphological analyses

The sex was determined according to the presence/absence of a penis. Genus *Heleobia* is dioecious, males develop a conspicuous penis on the right side of the head, ornamented with apocrine glands or accessory lobes^10,18–21,81–83^. In the mudsnail the penis is simple (i.e., devoid of apocrine glands or lobes^23,32,71,84^), colorless or whitish, mostly with an elongate spot of black pigment. However, non-native mudsnail populations are composed typically of parthenogenetic females, with males being rare or absent^23,43,85,86^. Genus *Potamolithus* is dioecious, the penis is simple, variable in pigmentation among taxa^87–92^; the females of some species develop a nuchal node^88–90^. In contrast to *Heleobia* and *Potamolithus*, whose species are oviparous^18,19,82,93,94^, the mudsnail is ovoviviparous, with females brooding small juvenile in the pallial oviduct^25,26,32,33,41^. To determine the presence of embryos or juveniles in the brood pouches, the oviduct wall was dissected and examined using a stereomicroscope. To observe the radula, the buccal mass was put in a dilute sodium hypochlorite solution for 3–10 minutes to isolate the structure and remove the organic material to subsequently be mounted on specimen stubs and observed using scanning electron microscopy (SEM) (Hitachi SU3500; Jeol JSM–6610LV). Similarly, the operculum was isolated from the foot and treated in the same way as the radula. The protoconch of specimens collected was also immersed in a sodium hypochlorite solution and observed using SEM. The shell of the type specimens of Truncatelloidean taxa described from the studied area housed at the MNHNCL^13,14^ were examined using stereomicroscope; the protoconch was observed under light microscope. The observations were compared with data from the literature^10,23,32,89,92,95,96^.

### Molecular analysis

DNA extraction, amplification and sequencing of partial DNA sequences of the mitochondrial gene cytochrome c oxidase subunit I (*COI*) ranging from 511 to 638 bp were performed following Collado^25^. The sequences were edited and aligned in BioEdit^97^ and then analyzed together with homologous sequences of related taxa accessed through GenBank. The COI matrix was used to generate a Bayesian tree with the MrBayes v. 3.1.2 program^98^ based on the HKY + I + G model of sequence evolution identified as the best-fitting substitution model in jModelTest^99^ using the Akaike Information Criterion. The analysis was run for 5,000,000 generations, sampling every 1,000 generations, and setting a burn-in period of 20%. The node support was estimated as Bayesian posterior probabilities (BPP). The species *Ascorhis tasmanica* (Martens, 1858) was used to root the tree. Average p-distances among sequences were obtained in Mega 7^100^.

## Supplementary information

Table S1-S3

## Data Availability

The sequences obtained in the present study were deposited in GenBank (accession numbers MH729593-MH729628) (Table S2). Voucher specimens were deposited in the Museo Nacional de Historia Natural (MNHNCL), Santiago, Chile, Museo de Zoología de la Universidad de Concepción (MZUC–UCC), Concepción, Chile, and Museo de Ciencias Naturales y Arqueología “Profesor Pedro Ramírez Fuentes”, Chillán, Chile (Table S3).
