# Supplementary material for: Morphological and molecular analysis of cryptic native and invasive freshwater snails in Chile
Source: Sci Rep. 2019 May 24;9:7846. doi: 10.1038/s41598-019-41279-x (PMC6534575; doi:10.1038/s41598-019-41279-x)
Supplement: Supplementary file 1 — Table S1-S3 [file 41598_2019_41279_MOESM1_ESM.pdf]

Morphological and molecular analysis of cryptic native and invasive freshwater snails in Chile

Gonzalo A. Collado[1,2], Marcela A. Vidal[1,2], Karina P. Aguayo[1], Marco A. Méndez[3], Moisés A. Valladares[3], Francisco J. Cabrera[4], Luis Pastenes[5], Diego E. Gutiérrez Gregoric[6,7] & Nicolas Puillandre[8]

[1]Departamento de Ciencias Básicas, Facultad de Ciencias, Universidad del Bío-Bío, Chillán, Chile.

[2]Grupo de Biodiversidad y Cambio Global, Universidad del Bío-Bío, Chillán, Chile.

[3]Laboratorio de Genética y Evolución, Facultad de Ciencias, Universidad de Chile, Santiago, Chile.

[4]Universidad Tecnológica de Chile INACAP, Chillán, Chile.

[5]Departamento de Biología y Química, Facultad de Ciencias Básicas, Universidad Católica del Maule, Talca, Chile.

[6]División Zoología Invertebrados, Museo de La Plata, Facultad de Ciencias Naturales y Museo Universidad Nacional de La Plata, La Plata Buenos Aires, Argentina.

[7]Consejo Nacional de Investigaciones Científicas y Técnicas (CONICET), CCT La Plata, Buenos Aires, Argentina

[8]Institut Systématique Evolution Biodiversité (ISYEB), Muséum national d'Histoire naturelle, CNRS, Sorbonne Université, EPHE, 57 rue Cuvier, CP 26 75005, Paris, France.

Table S1. Sampling sites, sexuality and presence/absence of brood pouches in truncatelloidean snails sampled in central Chile. The letter *a* denotes type locality; *b*: locality called Chalinga River in previous studies[18,24]; *c:* samples of previous studied restudied[18,24,25,Collado et al. in press]; Nt: non truncatelloidean snails.

**Basin/Locality**	Latitude (S)	Longitude (W)	**Snail (n)**	**Sex**	**Nuchal lobe**
Elqui Basin					
El Culebrón	29°57'38.57"	71°19'16.40"	*Heleobia* (20)	♂, ♀	No
Lagunillas Basin	30°07'7.74"	71°21'52.64"			
La Hacienda	30°07'7.74"	71°21'52.64"	*Heleobia* (20)	♂, ♀	No
Tongoy Basin					
Las Salinas	30°16'51.21"	71°30'17.07"	*Heleobia* (20)	♂, ♀	No
Limarí Basin					
Canal El Manzano	30°38'10.21"	71°16'59.99"	*Heleobia* (10)	♂, ♀	No
La Cebada	30°40'29.89"	71°32'50.72"	*Heleobia* (20)	♂, ♀	No
Caleta Toro	30°43'52.86"	71°40'4.18"	*Heleobia* (20)	♂, ♀	No
Cogotí River	31°05'6.42"	70°57'26.43"	Nt		
Combarbalá River	31°10'27.30"	70°59'54.81"	Nt		
Puente Pama	31°12'45.22"	71° 3'21.28"	Nt		
Choapa River Basin					
Choapa River [a]	31°46'58.1''	70°58'27.3''	*P. antipodarum* (10)	♀	No
Chalinga River					
Cunlagua	31°46'15.61"	70°59'05.09"	*P. antipodarum* (10)	♀	No
La Brunina [a]	31°44'23.20''	70°57'12.80''	*P. antipodarum* (20)	♀	No
Zapallar [a]	31°44'02.70''	70°53'17.80''	*P. antipodarum* (10)	♀	No
Canal Zapallar	31°42'32.90''	70°45'10.30''	*P. antipodarum* (10)	♀	No
Salamanca [a,b,c]	31°41'59.40''	70°45'13.00''	*P. antipodarum* (23)	♀	No
Camisas Stream	31°46'13.9''	71°03'57.90''	*P. antipodarum* (20)	♀	No
Consuelo Stream [a,c]	31°46'45.00''	70°57'47.30''	*P. antipodarum* (50)	♀	No
Illapel River	31°37'55.3''	71°09'20.30''	*P. antipodarum* (10)	♀	No
Limahuida Stream	31°45'58.00''	71°09'24.70''	*P. antipodarum* (20)	♀	No
Huentelauquén Norte	31°36'43.39"	71°31'29.29"	*P. antipodarum* (20)	♀	No

Huentelauquén Sur	31°37'16.06"	71°33'04.01"	*Heleobia* (20)	♂, ♀	No
Conchali Basin					
Laguna Conchalí	31°52'44.73"	71°29'48.36"	*Heleobia* (20)	♂, ♀	No
Quilimarí Basin					
Quilimarí River	32° 7'1.10"	71°30'37.73"	*Heleobia* (60)	♂, ♀	No
Pichidangui Basin					
Pichidangui	32° 8'19.64"	71°31'32.57"	*Heleobia* (20)	♂, ♀	No
Chivato Basin					
Los Molles	32°14'10.05"	71°30'28.11"	*Heleobia* (63)	♂, ♀	No
La Ballena Basin					
La Ballena	32°14'18.06"	71°29'53.03"	*Heleobia* (20)	♂, ♀	No
Cachagua Basin					
Cachagua	32°35'2.88"	71°26'52.02"	*Heleobia* (20)	♂, ♀	No
Laguna Zapallar Basin					
Laguna Zapallar	32°37'57.27"	71°25'34.02"	*Heleobia* (20)	♂, ♀	No
Puchuncavi Basin					
Ventanas	32°45'2.38"	71°28'40.68"	*Heleobia* (20)	♂, ♀	No
Petorca Basin					
Pedernal River	32° 7'57.23"	70°47'54.28"	Nt		
Chalaco	32°10'40.16"	70°48'10.34"	Nt		
El Sobrante River	32°13'56.56"	70°43'45.68"	*Potamolithus* (20)	♀	Yes
Chincolco	32°13'20.36"	70°50'07.11"	*P. antipodarum* (2)	♀	No
Ligua Basin					
Canal La Laja[c]	32°25'28.91"	71° 3'44.86"	*P. antipodarum* (30)	♀	No
Salinas de Pullally	32°25'28.02"	71°22'58.28"	*Heleobia* (20)	♂, ♀	No
Pichicuy	32°20'53.98"	71°26'34.02"	*Heleobia* (20)	♂, ♀	No
Longotoma	32°23'50.19"	71°23'02.66"	*Heleobia* (10)	♂, ♀	No

Aconcagua Basin					
San Rafael	32°48'1.41"	71° 3'29.00"	*Potamolithus* (10)	♀	?
Estero Romeral[c]	32°49'55.12"	71° 5'46.52"	*P. antipodarum* (20)	♀	No
Los Tilos Spring	32°47'31.14"	71° 4'26.88"	*Potamolithus* (14)	♀	No
Limache Stream	33° 0'23.57"	71°14'55.47"	Nt		
Puente Santa Julia	32°54’55.54”	71°29’57.60”	Nt		
Aconcagua River (Concón)	32°55'11.64"	71°30'14.99"	*Heleobia* (30)	♂, ♀	No
Las Palmas	-	-	Nt		
La Dormida	-	-	Nt		
Quebrada Escobares	33°04'52.22"	71°18'13.43"	*Potamolithus* (10)	♀	?
Marga Marga Basin					
El Salto	33°03’40.04”	71°28’23.50”	Nt		
Estero Quilpué	33°04’59.43”	71°36’00.59”	Nt		
Curauma Basin					
Las Docas	33°08’24.14”	71°42’20.23”	*Heleobia* (40)	♂, ♀	No
Maipo Basin					
Dehesa Stream [a,c]	33°22’02.00”	70° 31’15.00”	*P. antipodarum* (20)	♀	No
Parque O’Higgins Spring[c]	33°28’06.22”	70°39’38.31”	*P. antipodarum* (50)	♀	No
El Yeso Spring[c]	33°48’34.67”	70°12’37.00”	*P. antipodarum* (30)	♀	No
			Potamolithus (30)	♀	Yes
Altos de Cantillana	33°50’43.20”	70° 59’53.34”	*Potamolithus* (10)	♂, ♀	Yes
El Yali Basin					
Humedal El Yali	-	-	*Heleobia* (10)	♂, ♀	No
Rapel Basin					
Lo Carreño[c]	34°36'45.00"	70°55'47,8"	*P. antipodarum* (20)	♀	No
			Potamolithus (10)	♀	Yes
Vichuquén Basin					

Iloca	34°57'01.16"	72°11'04,05"	*Heleobia* (10)	♂, ♀	No
Laguna Torca	34°46'41.12"	72°02'41,72"	*Heleobia* (10)	♂, ♀	No
Mataquito Basin					
Guaico Spring	-	-	*Potamolithus* (10)	♀	Yes
Maule Basin					
Talca Chico[c]	35°27’30.98”	71° 42’58.62”	*Potamolithus* (10)	♀	Yes
Viña Casas Maule[c]	35°33’04.37”	71°40’12.25”	*Potamolithus* (6)	♀	Yes
El Colorado[c]	-	-	*Potamolithus* (20)	♀	Yes

Table S2. GenBank accession numbers (GB n) of COI sequences used in the phylogenetic analysis.

Species	GB n	Location	Source
Ascorhis tasmanica (Martens, 1858)	AF129329	Australia	Hershler *et al*. (1999)
Semisalsa stagnorum (Gmelin, 1791)	JQ973024	Zierikzee, Netherlands	Kroll *et al*. (2012)
Heleobops carrikeri Davis and McKee, 1989	JQ973018	Falmouth, U.S.	Kroll *et al*. (2012)
Heleobia australis (d'Orbigny, 1835)	JQ972708	Mar Chiquita, Argentina	Kroll *et al*. (2012)
Heleobia sp.	JQ973060	Elqui River, Chile	Kroll *et al*. (2012)
Heleobia sp.	JQ973061	Limarí River, Chile	Kroll *et al*. (2012)
Heleobia cf. *cumingii* (d'Orbigny, 1835)	JQ973041	Aconcagua River, Chile	Kroll *et al*. (2012)
Heleobia carcotensis Collado *et al*., 2016	KR816829	Salar de Carcote, Chile	Collado *et al*. (2016a)
Heleobia ascotanensis (Courty, 1907)	KF658133	Salar de Ascotán, Chile	Collado *et al*. (2016b)
Potamopyrgus antipodarum (Gray, 1843)	KJ616603	Parque O'Higgins Spring, Chile	Collado (2014)
	KJ616608	Chalinga River, Chile	Collado (2014)
	KJ616611	Consuelo Stream, Chile	Collado (2014)
	KJ616615	Dehesa Stream, Chile	Collado (2014)
	AB703675	Chitose River, Japan	Hamada *et al*. (2013)
	MH536535/36	Lo Carreño, Chile	Collado *et al*. (in revision)
	MH729616	Choapa River	This study
	MH729617/18	Huentelauquén Norte, Chile	This study
	MH729619/20	La Brunina, Chile	This study
Potamopyrgus estuarinus Winterbourn, 1970	AY631103	Raglan, New Zealand	Haase (2005)
Potamopyrgus kaitunuparaoa Haase, 2008	AY631105	Mokau, New Zealand	Haase (2005)
Potamopyrgus oppidanus Haase, 2008	AY631112	Wadestown, New Zealand	Haase (2005)
Potamolithus lapidum supersulcatus Pilsbry,	KX158843	Río Uruguay, Argentina	de Lucía and Gutiérrez
Potamolithus elenae de Lucía and Gutiérrez	KX397599	Río Negro province, Argentina	de Lucía and Gutiérrez
"Heleobia" hatcheri (Pilsbry, 1911)	KM220905	San Juan, Argentina	Koch *et al*. (2015)
Heleobia piscium (d'Orbigny, 1835)	KM220906	Isla Martín García, Argentina	Koch *et al*. (2015)

Heleobia sp.	KM220908	Uspallata, Argentina	Koch *et al*. (2015)
Potamolithus buschii (Frauenfeld, 1865)	KM220909	Rio de La Plata, Argentina	Koch *et al*. (2015)
Potamolithus agapetus Pilsbry, 1911	KM220910	Isla Martín García, Argentina	Koch *et al*. (2015)
Potamolithus ribeirensis Pilsbry, 1911	JX970618	Sao Paulo, Brazil	Wilke *et al*. (2013)
Potamolithus santiagensis (Biese, 1944)	MH536524/25	El Yeso Spring, Chile	Collado *et al*. (in press)
	MH536526/27	Lo Carreño, Chile	Collado *et al*. (in press)
	MH536528	El Colorado, Chile	Collado *et al*. (in press)
Potamolithus sp.	MH536530	Viña Casas del Maule, Chile	Collado *et al*. (in press)
	MH536531/32	Puerto Chico, lago Llanquihue,	Collado *et al*. (in press)
	MH729621	Los Tilos, Chile	This study
	MH729622/23	San Rafael, Chile	This study
	MH729624/25	Quebrada Escobares, Chile	This study
	MH729626	Guaico Spring, Chile	This study
	MH729627/28	Talca Chico, Chile	This study
Heleobia sp.	MH729593/94	El Culebrón, Chile	This study
	MH729595/96	Caleta Toro, Chile	This study
	MH729597/98	La Cebada, Chile	This study
	MH729599/00	Huentelauquén Sur, Chile	This study
	MH729601/02	Laguna Conchalí, Chile	This study
	MH729603/04	Quilimarí, Chile	This study
	MH729605/06	Los Molles, Chile	This study
	MH729607/08	La Ballena, Chile	This study
	MH729609	Laguna Zapallar, Chile	This study
	MH729610/11	Concón, río Aconcagua, Chile	This study
	MH729612/13	Las Docas, Chile	This study
	MH729614/15	El Yali, Chile	This study

Tabla S3. Voucher specimens of Truncatelloidea of the present study housed in museums.

Species/Locality	UBB voucher*	Sample type	Museum specimen
Potamopyrgus antipodarum			
Huentelauquén (north sector of the town),	Norte 1 museo	Shell	MCNAPRF 139 – 01
Huentelauquén (north sector of the town),	Norte 2 museo	Shell	MCNAPRF 139 – 02
Huentelauquén (north sector of the town),	Norte 3 museo	Shell	MCNAPRF 139 – 03
Vertiente El Yeso, Cajón del Maipo, Región	4	Shell	MNHNCL 203744
Vertiente El Yeso, Cajón del Maipo, Región	38 to 42	Shells	MNHNCL 203745
Vertiente El Yeso, Cajón del Maipo, Región	6	Protoconch	MNHNCL 203746
Vertiente El Yeso, Cajón del Maipo, Región	6	Operculum	MNHNCL 203747
Vertiente El Yeso, Cajón del Maipo, Región	25	Operculum	MNHNCL 203748
Vertiente El Yeso, Cajón del Maipo, Región	23	Radula	MNHNCL 203749
Vertiente El Yeso, Cajón del Maipo, Región	1 museo	Shell	MZUC–UCC 43993
Vertiente El Yeso, Cajón del Maipo, Región	2 museo	Shell	MZUC–UCC 43994
Vertiente El Yeso, Cajón del Maipo, Región	3 museo	Shell	MZUC–UCC 43995
Vertiente El Yeso, Cajón del Maipo, Región	4 museo	Shell	MZUC–UCC 43996
Vertiente El Yeso, Cajón del Maipo, Región	5 museo	Shell	MZUC–UCC 43997
Vertiente El Yeso, Cajón del Maipo, Región	6 museo	Shell	MZUC–UCC 43998
Vertiente El Yeso, Cajón del Maipo, Región	7 museo	Shell	MZUC–UCC 43999
Vertiente El Yeso, Cajón del Maipo, Región	8 museo	Shell	MZUC–UCC 43000
Vertiente El Yeso, Cajón del Maipo, Región	9 museo	Shell	MZUC–UCC 44001
Vertiente El Yeso, Cajón del Maipo, Región	10 museo	Shell	MZUC–UCC 44002
La Brunina, Región de Coquimbo	Museo 1	Shell	MCNAPRF 151 – 01
La Brunina, Región de Coquimbo	6	Shell	MCNAPRF 151 – 02
La Brunina, Región de Coquimbo	3	radula	MCNAPRF 151 – 03
La Brunina, Región de Coquimbo	5	Operculum	MCNAPRF 151 – 04
La Brunina, Región de Coquimbo	6	Operculum	MCNAPRF 151 – 05
La Brunina, Región de Coquimbo	23	Operculum	MCNAPRF 151 – 06
La Brunina, Región de Coquimbo	10	Soft body	MCNAPRF 151 – 07

Zapallar, Chalinga River, Región de	6	Soft body	MCNAPRF 151 – 08
Camisas River (Corrales), Región de	1	Soft body	MCNAPRF 151 – 09
Camisas River (Corrales), Región de	4	Soft body	MCNAPRF 151 – 10
Limahuida Stream, Región de Coquimbo	8	Protoconchs (juvenile)	MCNAPRF 151 – 11
Limahuida Stream, Región de Coquimbo	9	Protoconch	MCNAPRF 151 – 12
***Potamolithus* sp.**			
San Rafael, Región de Valparaíso	1	Soft body	MCNAPRF 151 – 13
San Rafael, Región de Valparaíso	2	Soft body	MCNAPRF 151 – 14
San Rafael, Región de Valparaíso	11	Operculum	MCNAPRF 151 – 15
San Rafael, Región de Valparaíso	12	Radula	MCNAPRF 151 – 16
San Rafael, Región de Valparaíso	13	Protoconch	MCNAPRF 151 – 17
San Rafael, Región de Valparaíso	13	Operculum	MCNAPRF 151 – 18
San Rafael, Región de Valparaíso	13	Radula	MCNAPRF 151 – 19
El Sobrante River, Región de Valparaíso	19	Shell	MCNAPRF 151 – 20
El Sobrante River, Región de Valparaíso	19	Operculum	MCNAPRF 151 – 21
El Sobrante River, Región de Valparaíso	19	Radula	MCNAPRF 151 – 22
Quebrada Escobares, Región de Valparaíso	3	Protoconch	MCNAPRF 151 – 23
Talca Chico, Región del Maule	3	Radula	MCNAPRF 151 – 24
Perquelauquén, Región del Maule	1	Soft body	MCNAPRF 151 – 25
Licán Ray, Región de la Araucanía	2	Soft body	MCNAPRF 151 – 26
Licán Ray, Región de la Araucanía	3	Radula	MCNAPRF 151 – 27
Licán Ray, Región de la Araucanía	4	Radula	MCNAPRF 151 – 28
Licán Ray, Región de la Araucanía	4	Protoconch	MCNAPRF 151 – 29
El Sobrante, Petorca, Región de Valparaíso	17	Shell	MCNAPRF 139 – 04
El Sobrante, Petorca, Región de Valparaíso	18	Shell	MCNAPRF 139 – 05
San Rafael, Región de Valparaíso	11	Protoconch	MNHNCL 203739
San Rafael, Región de Valparaíso	14	Protoconch	MNHNCL 203740
San Rafael, Región de Valparaíso	15	Protoconch	MNHNCL 203741
San Rafael, Región de Valparaíso	12	Operculum	MNHNCL 203742
Potamolithus santiagensis			
El Yeso Spring, Región Metropolitana	37	Radula	MNHNCL 203743
***Heleobia* sp.**			
Humedal El Culebrón, Región de Coquimbo	11	Soft body, male	MCNAPRF 139 – 06

Las Salinas, Tongoy, Región de Coquimbo	3	Soft body, female	MCNAPRF 139 – 07
Las Salinas, Tongoy, Región de Coquimbo	15	Shell	MCNAPRF 139 – 08
Las Salinas, Tongoy, Región de Coquimbo	16	Shell	MCNAPRF 139 – 09
Ventanas, Región de Valparaíso	5	Soft body, female	MCNAPRF 139 – 10
El Manzano, Limarí, Región de Coquimbo	4	Operculum	MCNAPRF 151 – 30
Caleta Toro, Limarí River, Región de	6	Protoconch	MCNAPRF 151 – 31
Huentelauquén (south sector of the town),	4	Protoconch	MCNAPRF 151 – 32
Huentelauquén (south sector of the town),	4	Radula	MCNAPRF 151 – 33
Huentelauquén (south sector of the town),	8	Protoconch	MCNAPRF 151 – 34
Huentelauquén (south sector of the town),	8	Operculum	MCNAPRF 151 – 35
Huentelauquén (south sector of the town),	8	Radula	MCNAPRF 151 – 36
Quilimarí, Región de Coquimbo	68	Operculum	MCNAPRF 151 – 37
Quilimarí, Región de Coquimbo	69	Operculum	MCNAPRF 151 – 38
Los Molles, Región de Valparaíso	63	Shell	MCNAPRF 151 – 39
Pichicuy, Región de Valparaíso	7	Protoconch	MCNAPRF 151 – 40
Ventanas, Región de Valparaíso	11	Protoconch	MCNAPRF 151 – 41
Los Molles, Región de Valparaíso	61	Shell	MNHNCL 203733
La Ballena, Región de Valparaíso	13	Protoconch	MNHNCL 203734
La Ballena, Región de Valparaíso	16	Protoconch	MNHNCL 203735
Ventanas, Región de Valparaíso	6	Radula	MNHNCL 203736
Ventanas, Región de Valparaíso	12	Radula	MNHNCL 203737
Ventanas, Región de Valparaíso	6	Operculum	MNHNCL 203738

*: Data base of the Laboratorio de Malacología y Sistemática Molecular, Universidad del Bío-Bío (Campus Fernando May, Chillán, Chile).
